# Olfactory Science and Technology in Prostate Cancer Diagnosis: From Invertebrate Models to Artificial Intelligence

**DOI:** 10.3390/life16050848

**Published:** 2026-05-20

**Authors:** Mohamed A. A. A. Hegazi, Marta Noemi Monari, Fabio Pasqualini, Sara Beltrame, Chiara Martella, Carmen Bax, Lorenzo Tidu, Laura Maria Capelli, Gianluigi Taverna, Fabio Grizzi

**Affiliations:** 1 Department of Immunology and Inflammation, IRCCS Humanitas Research Hospital, Rozzano, 20089 Milan, Italy; lunamohamed75@gmail.com (M.A.A.A.H.); fabio.pasqualini@hunimed.eu (F.P.); 2 Laboratory Analysis, Humanitas Mater Domini, Castellanza, 21053 Varese, Italy; marta_noemi.monari@humanitas.it (M.N.M.); sara.beltrame@materdomini.it (S.B.); chiara.martella@materdomini.it (C.M.); 3 Department of Chemistry, Materials and Chemical Engineering “Giulio Natta”, Politecnico di Milano, 20133 Milan, Italy; carmen.bax@politecnico.it (C.B.); laura.capelli@polimi.it (L.M.C.); 4 Azienda Socio-Sanitaria di Cagliari, 09126 Cagliari, Italy; lorudit@virgilio.it; 5 Department of Urology, Humanitas Mater Domini, Castellanza, 21053 Varese, Italy; gianluigi.taverna@humanitas.it; 6 Department of Biomedical Sciences, Humanitas University, Pieve Emanuele, 20072 Milan, Italy

**Keywords:** prostate cancer, diagnosis, volatile organic compound, eNose, artificial intelligence

## Abstract

Prostate cancer (PCa) is one of the leading causes of cancer-related morbidity and mortality in men worldwide, and early detection remains crucial for ensuring effective treatment and improving patient outcomes. In this context, the development of non-invasive, accurate, and cost-effective screening strategies is of paramount importance. One particularly promising and innovative approach is the analysis of volatile organic compounds (VOCs), a field known as volatolomics. VOCs, which are metabolic by products released by the body, reflect underlying biochemical processes and offer a valuable, non-invasive source of diagnostic information. Recent advances have highlighted the potential of VOC profiling in PCa detection. A variety of biological systems have demonstrated remarkable sensitivity and specificity in recognizing disease-associated VOC signatures. Notably, trained dogs, selected invertebrates, and artificial sensing platforms have all shown the ability to identify PCa-related olfactory patterns. Among technological approaches, electronic noses (eNoses), which combine chemical sensor arrays with pattern recognition algorithms such as neural networks, represent a rapidly evolving diagnostic tool. Together, these biologically inspired and technology-driven strategies are reshaping the landscape of cancer diagnostics. They offer a compelling foundation for the development of rapid, non-invasive, and clinically translatable methods for PCa detection. This narrative review summarizes recent advances in using VOCs for PCa diagnosis and evaluates the reproducibility and clinical robustness of these approaches, focusing on challenges such as standardizing sampling, storage, and analysis, small cohort sizes, and the need for external validation and regulatory integration.

## 1. Introduction

Prostate cancer (PCa) is the most common cancer diagnosis in men, accounting for 30% of male cancers in 2025, and is the second leading cancer death in men behind lung cancer [1]. With the introduction of prostate-specific antigen (PSA) testing, the presentation of PCa has changed markedly, with most men now being diagnosed at a potentially curable stage. However, early detection also carries the risk of overdiagnosis, identifying small, indolent tumors that may never become clinically significant [2]. In such cases, active treatments such as surgery or radiation can significantly impair urinary, sexual, and bowel function, leading to a decline in quality of life [3,4].

The role of PSA testing in early detection has become increasingly debated [5], as growing evidence indicates its contribution to overdiagnosis and overtreatment. The specificity of PSA as a screening tool for PCa is limited, particularly at PSA levels below 10 μg/L [6]. This underscores the need for more accurate biomarkers to distinguish low- from high-risk disease, reducing unnecessary biopsies and associated psychological burden [7].

Although several biomarkers are currently available, including urinary (PCA3, SelectMDx), blood-based (4Kscore, PHI), and tissue-based (ConfirmMDx) tests, comparative clinical studies evaluating their performance and real-world utility remain limited [8].

PCa is widely recognized as a multifocal disease, characterized by the presence of multiple, genetically distinct histological tumor foci within the primary lesion [9]. Tumorigenesis is thought to progress from prostatic intraepithelial neoplasia (PIN) and/or atypical small acinar proliferation (ASAP) through localized and locally advanced stages, culminating in metastatic disease [10].

The diagnostic pathway for PCa comprises several stages, including initial patient presentation, the acquisition and interpretation of multiparametric magnetic resonance imaging (mpMRI) and the identification of suspicious target lesions [11]. This is followed by the biopsy procedure and subsequent evaluation of the tissue cores. The formal diagnosis of PCa is ascertained through microscopic scrutiny and involves assigning a Grade Group or Gleason score, as indicated by existing medical guidelines [12,13]. Following diagnostic confirmation, patients undergo risk stratification, employing various established algorithms such as D’Amico [14], International Society of Urological Pathology (ISUP) [15], National Comprehensive Cancer Network (NCCN) [16], and Cancer of the Prostate Risk Assessment (CAPRA) [17] for categorizing the risk.

According to the Early Detection of Prostate Cancer: AUA/SUO Guideline (2026) [18], which provides a framework for clinical decision-making in PCa screening and follow-up, several key recommendations are highlighted [19]. First, PSA testing remains the preferred first-line screening tool, supported by randomized evidence demonstrating reductions in metastatic disease and PCa mortality. In contrast, digital rectal examination (DRE) is not recommended as an initial screening test in asymptomatic individuals, though it may serve as a diagnostic tool in symptomatic patients. In parallel, the current European position emphasizes individualized, risk-adapted early detection in well-informed men, with PSA remaining the initial test [18]. In asymptomatic men with PSA values between 3 and 10 ng/mL and no suspicious findings on palpation, repeat PSA testing is recommended before proceeding to further diagnostic investigations, since PSA variability may otherwise lead to unnecessary downstream procedures [18]. In men with PSA values between 3 and 20 ng/mL, the indication for prostate biopsy should be refined using prostate MRI, calibrated risk calculators adapted to the target population, and/or selected additional serum or urine biomarker tests [18]. mpMRI has demonstrated clear clinical value in guiding biopsy decisions, improving detection of clinically significant disease while reducing overdiagnosis of indolent cancers. mpMRI has enabled a shift from transrectal ultrasound-guided 12-core systematic biopsies to newer techniques, such as MRI–ultrasound fusion biopsy, in which suspicious lesions on MRI can be specifically targeted during biopsy to reduce the likelihood of missed cancer [2]. Evidence from the PROBASE trial supports the role of imaging-based triage, while also emphasizing that diagnostic accuracy depends heavily on high-quality imaging and expert interpretation [20].

At the same time, emerging modalities such as 29 MHz micro-ultrasound (MUS) are being explored as potentially transformative tools in opportunistic PCa screening and have shown comparable diagnostic promise. However, no imaging modality to date has demonstrated an improvement in long-term outcomes, such as cancer-specific mortality. Evidence from the PROBASE trial, which included men aged 45 years, indicates that incorporating MRI following a PSA threshold of 3 ng/mL could reduce unnecessary biopsies by approximately 20% when using Prostate Imaging-Reporting and Data System (PI-RADS) scores of 3–5 as the threshold, and by up to 68% when applying a stricter cutoff of PI-RADS 4–5. This reduction, however, comes at the cost of missing approximately 13% of clinically significant PCa (csPCa) cases [21].

Although imaging-based triage is increasingly important in the diagnostic pathway, MRI should not be presented as a stand-alone initial screening tool. Current European recommendations explicitly state that MRI should not be used as an initial screening test, while recommending MRI before prostate biopsy in men with suspected organ-confined disease [18]. Therefore, MRI should be interpreted as part of a structured diagnostic algorithm following clinical and biochemical risk assessment, rather than as a replacement for PSA-based early detection. Despite technological advances showing that MR imaging can reduce overdiagnosis while preserving sensitivity for clinically significant disease, important limitations remain. These include missed or under-graded significant cancers due to imaging inaccuracies or sampling difficulties, as well as variability between observers in image interpretation [13,22]. Additionally, this notion is limited by the widespread availability and reimbursement of prostate MRI in many countries [21]. These issues are driving ongoing innovation in prostate imaging, including simplified MRI protocols and artificial intelligence (AI)-assisted analysis [23]. Additionally, current guidelines support the use of adjunctive urine or serum biomarkers in selected cases where further risk stratification may influence the decision to proceed with biopsy [18].

The identification and integration of novel biomarkers into clinical practice remain essential in PCa for improving detection, prognostication, and disease monitoring. They include traditional markers and emerging tools such as genetic, molecular, and protein assays, along with advanced imaging techniques [24]. These next-generation biomarkers support personalized risk assessment and treatment decisions, with ongoing advances expected to further improve clinical decision-making and prognostic accuracy [25,26].

An innovative and very promising approach that could meet these challenges is the study of chemical processes involving profiles of highly and semi-volatile organic compounds (VOCs), named “volatolomics” [27,28,29,30,31,32,33]. VOCs emitted by the human body may help reshape non-invasive diagnostic approaches [34,35,36,37]. Disease-specific VOCs are generated through distinct biochemical pathways and can be detected in biological fluids [31,38]. VOCs in breath, urine, and tissues can reflect underlying biological processes, with certain profiles associated with specific medical conditions [32,39,40,41]. The identification of VOC-based biomarkers for cancer detection represents a promising and rapidly advancing area of research. Both biological systems, including trained dogs and selected invertebrates, as well as artificial sensing platforms, have demonstrated the ability to discriminate PCa-related olfactory signatures. Among these technologies, electronic noses (eNoses), which integrate chemical sensor arrays with pattern recognition algorithms such as neural networks, are emerging as particularly promising diagnostic tools. Collectively, these biologically inspired and technology-driven approaches are contributing to a shift toward rapid, non-invasive and potentially clinically translatable methods for PCa detection. Although current clinical pathways for PCa detection continue to rely mainly on PSA testing, risk-adapted screening strategies, and mpMRI as a pre-biopsy triage tool, additional serum or urine biomarkers may be used as adjuncts in selected clinical contexts. In contrast, emerging approaches based on VOCs, including eNose systems, canine olfaction, and invertebrate models, remain investigational and are not yet incorporated into clinical guidelines for routine screening, biopsy decision-making, or risk stratification. This narrative review aims to summarize current advances in the use of VOCs for PCa diagnosis and to critically evaluate the reproducibility and clinical robustness of VOC-based approaches, with particular focus on challenges related to standardization of sampling, storage, and analytical procedures, small cohort sizes, and the need for external validation and regulatory integration.

## 2. *C. elegans* and the Diagnosis of Prostate Cancer

It has been reported that the small soil nematode *Caenorhabditis elegans* (*C. elegans*) possesses a highly developed chemosensory system and can detect a wide variety of volatile and water-soluble chemicals associated with food, danger, pathogens, or other organisms [42,43,44]. Chemosensation, encompassing the chemical senses of olfaction (smell) and gustation (taste), is an ancient biological process fundamental to survival that represents the primary sensory modality in this organism, enabling it to respond to environmental chemical cues that regulate essential behaviors such as feeding, avoidance of toxic compounds, and social interactions [45,46,47,48]. It refers to the transduction of chemical stimuli from the environment into neurological signals that can be interpreted by an organism [49]. Chemosensory systems enable direct interaction with environmental chemical cues and regulate behaviors essential for survival and reproduction, including foraging, predator avoidance, recognition of conspecifics, parental care, and mate attraction [49].

Despite having a relatively simple nervous system of only 302 neurons [42], *C. elegans* exhibits remarkable olfactory sensitivity and discrimination, supported by diverse chemosensory receptors and signaling pathways [50]. Its short lifespan, small size (~1 mm), hermaphroditic reproduction, and ease of cultivation make it a widely used model in biomedical research [51,52,53,54,55]. It can be maintained on agar plates with bacterial food and reaches adulthood within 3–4 days, enabling large-scale and high-throughput studies [56].

Its fully mapped connectome and extensive genetic tools make it especially valuable for studying sensory biology and metabolic signaling. The nervous system is largely devoted to chemosensation [54,57], with a significant portion of the genome encoding chemosensory receptors. Notably, at least 1500 predicted G protein-coupled receptors (GPCRs) function as olfactory receptors detecting volatile and soluble cues [58]. These receptors are expressed in amphid sensory neurons (AWA, AWB, AWC) in the head. Their activation triggers neural circuits regulating chemotaxis, guiding movement toward attractive stimuli and away from harmful ones. Signal transduction is mediated by G-protein pathways, particularly the ODR-3 Gα protein, essential for olfactory processing. This system enables detection of complex odor mixtures and coordinated behavioral responses [59].

**Figure 1:**
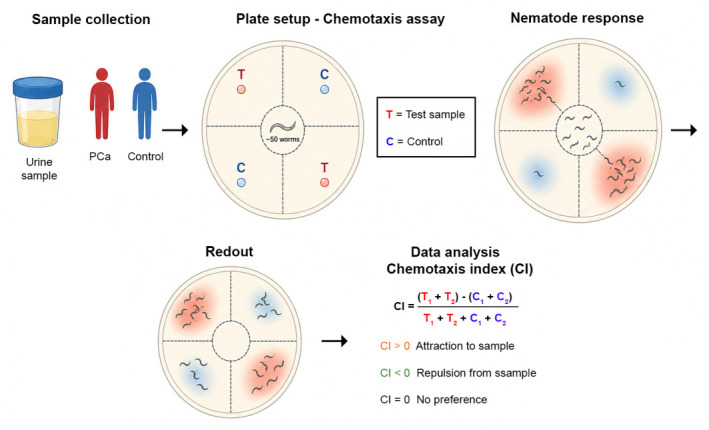
Schematic representation of the nematode-based chemotaxis assay used to evaluate the ability of *C. elegans* to detect VOCs associated with PCa. Urine samples from PCa patients and controls are applied to agar plates in opposing quadrants (test, T) with control diluent (control; C) placed in the remaining quadrants. Nematodes migrate in response to chemical cues via their chemosensory system, accumulating preferentially in attractive or avoiding repulsive regions. After incubation, plates are imaged and worms in each quadrant are counted. Chemotactic behavior is quantified using a chemotaxis index (CI) calculated as $CI\;=\;\left(T^{1}+\;T^{2}\right)-\frac{\left(C^{1}+\;C^{2}\right)}{\left(T^{1}+\;T^{2}\right)}+\left(C^{1}+\;C^{2}\right)$, where positive values indicate attraction, negative values indicate repulsion, and CI = 0 indicates no preference [62,63]. Nematodes placed at the center respond to chemical cues via their chemosensory system, migrating toward or away from the stimuli.

Recent research has demonstrated that this highly sensitive olfactory system can also detect chemical signatures associated with human diseases, including cancer. In particular, Hirotsu et al. developed a nematode-based cancer detection method known as the Nematode Scent Detection Test (NSDT), which exploits the ability of *C. elegans* to respond to cancer-associated odorants present in biological samples such as urine [58]. The test is based on a simple chemotaxis assay in which nematodes are exposed to diluted human urine samples on agar plates (Figure 1). When presented with urine derived from cancer patients, *C. elegans* typically exhibits strong attraction, whereas urine from healthy individuals induces avoidance behavior. Using this assay system, Hirotsu et al. demonstrated that *C. elegans* can detect multiple cancer types, including colorectal, gastric, breast, and PCa, with remarkable diagnostic performance [58].

In their study involving human urine samples, the NSDT achieved a sensitivity of approximately 95.8% and a specificity of around 95% [58]. Importantly, the assay was able to detect cancers even at early stages (stage 0-I), suggesting that cancer-related metabolic alterations produce detectable VOC signatures before clinical symptoms appear. Subsequent studies have confirmed that the nematode chemotaxis assay can detect a variety of malignancies beyond the tumor types initially investigated [60,61]. In these studies, the nematode-based assay achieved sensitivities of up to 96–100% for certain tumor types, further supporting the potential of this biological sensing system as a non-invasive diagnostic tool [59].

The chemotaxis assay underlying this detection system is widely used in *C. elegans* behavioral research and quantifies the relative attraction or repulsion of worms toward specific chemical cues. In this experimental setup, worms are placed at the center of an agar plate containing two opposite spots: one with the test sample (e.g., diluted urine) and one control spot. After a defined period, typically 30–60 min, the distribution of nematodes on the plate is quantified and expressed as a chemotaxis index (Figure 1). Positive values indicate attraction toward the sample, while negative values indicate avoidance behavior [56]. The mechanism underlying this phenomenon is thought to involve cancer-associated VOCs present in urine. Cancer cells undergo profound metabolic alterations that lead to the production of characteristic metabolites and degradation products that are excreted in body fluids. These metabolic signatures contribute to the so-called urinary volatilome, a complex mixture of volatile compounds that can differ significantly between healthy individuals and cancer patients [33,50]. Because *C. elegans* possesses extremely sensitive olfactory receptors capable of detecting odorants even at nanomolar concentrations, the nematode can discriminate subtle differences in chemical composition that may be difficult to identify using conventional analytical techniques. It is known that cancer-associated volatilome comprises diverse chemical classes, including aldehydes, organic acids, hydrocarbons, benzene derivatives, terpenes, and phenolic compounds [33]. These metabolites arise from cancer-related metabolic reprogramming, such as oxidative stress, lipid peroxidation, and altered enzymatic activity. As they are excreted into biological fluids, including urine, they represent potential non-invasive biomarkers for cancer detection [59]. In 2021, Thompson et al. demonstrated that *C. elegans* can specifically distinguish urine samples from PCa patients and those from cancer-free controls using a similar chemotaxis-based behavioral assay [63] (Table 1). In their study, diluted urine samples collected from men undergoing PCa screening were tested using young adult nematodes of the N2 strain. The results showed that *C. elegans* displayed a significant attraction to urine from PCa patients, whereas urine from benign or negative-screen individuals elicited neutral or repulsive responses. Interestingly, the study also showed that the behavioral response of *C. elegans* was dilution-dependent, with optimal discrimination observed at a 1:100 dilution of urine samples. When the chemotaxis index derived from the nematode assay was combined with serum PSA levels, the overall accuracy of patient classification improved significantly, reaching approximately 81%. This combined diagnostic approach also improved specificity compared with PSA testing alone, suggesting that nematode-based scent detection could potentially reduce false-positive rates and unnecessary prostate biopsies [63]. More recently, the concept of using *C. elegans* as a biosensor has been proposed within the broader framework of multi-cancer early detection (MCED) strategies. The nematode-based strategy may eventually serve as a complementary tool to existing diagnostic methods by potentially enhancing the detection of cancer-associated metabolic signatures in urine and other biological fluids, pending further confirmation of its sensitivity, specificity, and reproducibility.

Although these models remain at an early and experimental stage of development, the proposed approach may offer a potential alternative or complement to conventional biomarker-based screening strategies. In the long term, it could contribute to ongoing research aimed at developing more universal cancer screening tools [64]. As a non-invasive, rapid, simple, and potentially cost-effective assay, this approach has attracted growing interest as a candidate screening method for the early detection of cancers beyond [65,66,67]. However, its clinical utility remains to be established through rigorous validation studies confirming sensitivity, specificity, and reproducibility before any clinical application can be considered. In this context, the nematode-based test known as N-NOSE has been developed as a urine-based screening method capable of detecting multiple cancer types simultaneously, including PCa [56].

## 3. Canine Olfactory System and the Diagnosis of Prostate Cancer

In recent decades, detection dogs, species with greater levels of olfactory function (i.e., microsmatic [68]) have been widely considered the most effective and adaptive method to detect explosives, narcotics, and other illegal materials [69,70]. The canine olfactory system is well-adapted to the detection of a vast number of odorous substances that differ in magnitude, volatility and concentration [71] as well as molecules showing small differences in stereoisomeric structure [69,72,73,74]. Dogs are widely employed in law enforcement for detecting explosives, drugs, and missing persons due to their exceptional olfactory sensitivity, which enables them to perceive compounds at concentrations as low as parts per trillion [75]. This remarkable capability is supported by specific anatomical features, including a highly developed olfactory epithelium, a large repertoire of olfactory receptors, and dense innervation of the olfactory mucosa [75]. It has been shown that dogs can detect cancer by odor at early stages; moreover, the reliability of dogs is higher than that of electronic sensors for detecting volatile tumor markers. These characteristics have stimulated interest in their application to medical diagnostics, particularly for the detection of PCa-related VOCs in biological samples such as urine (Table 2).

The studies summarized in Table 2 consistently demonstrate that dogs can be trained to recognize patterns of VOCs. In a study employing dogs to detect PCa from urine samples, Taverna et al. [80] described a structured training protocol comprising four sequential phases. This protocol progressively increased the heterogeneity of control samples, beginning with healthy young individuals and gradually incorporating both healthy and diseased subjects of different sexes and age groups.

**Figure 2:**
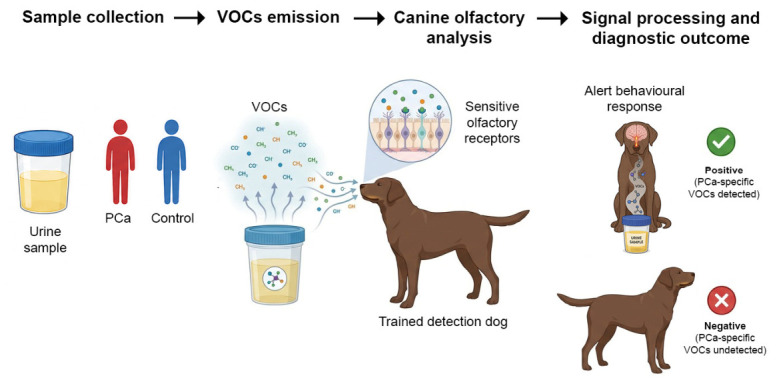
Schematic overview of the experimental workflow for PCa detection using trained detection dogs based on VOCs. Urine samples are collected from PCa patients and healthy controls, each characterized by distinct VOC profiles. In the diagnostic workflow, dogs are exposed to multiple urine samples in a blinded setting and indicate PCa-positive or control samples through trained behavioral responses, such as sitting or stopping in front of the sample after olfactory inspection. Training and testing phases of detection dogs: during training, dogs learn to associate PCa-related odors with positive reinforcement; during testing, they are presented with unknown samples and discriminate between PCa-associated and control odors without prior information. This approach exploits the high sensitivity of the canine olfactory system to disease-related VOC signatures and supports its potential application in non-invasive cancer detection.

Throughout all training phases, task complexity was systematically enhanced by adjusting the ratio of positive to negative samples and by introducing randomized sets of six samples containing varying numbers of PC-positive specimens (Figure 2). To ensure robustness and minimize conditioned responses, the protocol included negative control runs, randomized sample positioning, and a requirement that only detections consistently reproduced across repeated trials were considered valid. Although animals’ olfactory abilities were well known and widely used, their role in cancer detection was not explored until the late 1980s, when anecdotal reports described unusual dog behavior toward owners with tumors. The first study, a 1989 case report, described a 44-year-old woman whose skin lesion, later diagnosed as malignant melanoma, was identified after her Border Collie and Doberman Pinscher repeatedly sniffed it [83]. Early investigations into canine detection of PCa produced heterogeneous results. Gordon et al. [76] reported relatively low sensitivity, around 20%, despite moderate specificity, likely due to limitations in training protocols, including the absence of standardized procedures and professional handling. In contrast, Cornu et al. [77] demonstrated markedly improved performance, with sensitivity and specificity reaching approximately 91% following an extended and structured training period using a Belgian Malinois. Similarly, Taverna et al. [80] implemented a rigorous and standardized training protocol in a large cohort of over 900 participants, achieving sensitivity greater than 98% and specificity above 96%, supporting the hypothesis that dogs can reliably detect PCa-associated VOC signatures (Figure 2). These findings have been subsequently supported by Guest et al. and others [81,84,85].

These promising findings prompted further investigation into the ability of highly trained dogs to detect PCa biochemical recurrence (BCR) in patients who had previously undergone radical prostatectomy [86]. The results demonstrate that trained dogs are also capable of identifying PCa BCR. In 2024, Hermieu et al. [82] reported that canine olfaction, as a non-invasive and safe approach, appears to be a reliable method for detecting ISUP ≥ 2 PCa. [82] When combined with prostate MRI, it may further enhance clinical decision-making regarding the need for prostate biopsy. However, not all studies have confirmed such high diagnostic accuracy. Elliker et al. [78] reported lower sensitivity and specificity compared with other studies, which may be attributable to methodological limitations. In particular, the repeated exposure of dogs to the same training samples may have led to overfitting to specific odor signatures rather than learning generalizable cancer-associated VOC patterns. Consequently, the dogs’ ability to correctly classify novel, unseen samples could have been compromised. This limitation highlights the importance of using diverse and independent training and validation datasets to ensure robust generalization. These findings also emphasize the need for standardized training protocols, controlled sample handling, and rigorous validation strategies to guarantee reproducibility and reliability in clinical settings [84,87,88,89]. Although canine olfaction represents a promising non-invasive approach for PCa detection, further standardization and large-scale validation remain necessary to support its clinical implementation [86,90,91]. Several practical constraints continue to limit routine adoption, including the requirement for specialized facilities, extensive training of both dogs and handlers, the limited working lifespan of dogs, and challenges in integrating such approaches into standardized clinical workflows [92,93]. While dogs and other animals exhibit remarkable olfactory detection capabilities, issues related to scalability, reproducibility, and standardization persist. Consequently, significant efforts have focused on developing technological systems that mimic biological olfaction. Devices such as the eNose aim to reproduce the sensitivity and pattern recognition abilities of animal olfactory systems using chemical sensors and advanced algorithms, offering a more standardized, scalable, and clinically applicable approach to cancer detection.

## 4. eNose and the Diagnosis of Prostate Cancer

**Figure 3:**
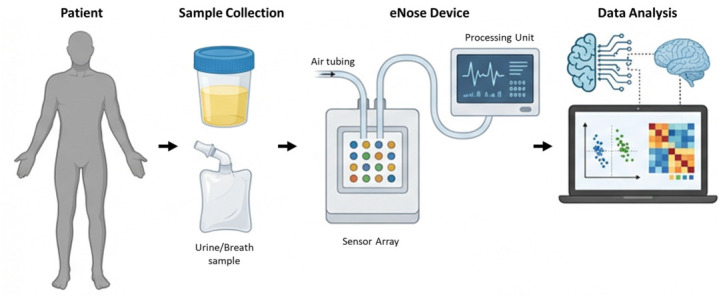
Schematic overview of eNose technology for PCa detection based on VOCs analysis. Biological samples (e.g., urine or breath) are collected from patients and introduced into the sensor system, where VOC mixtures interact with an array of chemical sensors. This interaction generates multidimensional electrical response patterns characteristic of the sample composition. The signals are transmitted to a processing unit, where pattern recognition and ML algorithms are applied to extract relevant features and classify samples according to disease status. This approach enables rapid, non-invasive analysis without the need for identification of individual compounds, relying instead on global VOC signatures associated with PCa.

According to the US Food and Drug Administration (FDA), dogs and other animals do not fall under the category of “devices,” and are thus ineligible for approval in clinical settings (https://www.fda.gov/medical-devices, accessed on 4 March 2026). This limitation accentuates the urgency for a technologically sophisticated alternative that can replicate the olfactory capabilities of animals like canines. The essential criteria for such an alternative would encompass its potential for reproducibility, accessibility, eligibility for FDA approval, and adaptability for clinical uses. Traditionally, VOCs have been measured using analytical instruments such as gas chromatography–mass spectrometry (GC–MS), which are unsuitable for diagnostic purposes due to high cost and procedural complexity [85]. eNoses are biomimetic devices engineered to emulate the sense of smell through arrays of chemical sensors. Initially developed for quality control and aroma profiling in the food and beverage sector, their use has since broadened to areas such as environmental monitoring and medical diagnostics. Notably, their capacity to identify and interpret complex chemical signatures in biological samples has positioned them as promising tools for non-invasive cancer detection [30]. The eNose has been proposed as a valid alternative approach for assessing PCa-specific VOCs (Table 3) [94,95]. Its core operating principle relies on an array of chemical sensors, typically composed of materials such as metal oxides, polymers, or conductive composites, with either broad or selective sensitivity. These sensors are integrated with machine learning (ML) and pattern recognition algorithms to interpret complex gaseous mixtures (Figure 3).

The eNose sensors exhibit changes in electrical and/or physical properties like resistance or capacitance, oscillation frequency or mass when exposed to different types of VOCs. One of the key advantages of eNoses is their ability to analyze complex mixtures without the need for separation, unlike techniques such as GC-MS. This makes them suitable for rapid, real-time analysis in a wide range of applications, from food quality control and environmental monitoring to medical diagnostics and military security [96,97,98,99], as well as in pharmaceutical and microbiology analyses [98,100]. Moreover, eNoses are generally more compact, portable, and cost-effective compared to traditional analytical methods. However, there are challenges that accompany the use of eNose. Sensor drift due to aging, sensitivity to environmental factors like temperature and humidity, and the need for frequent calibration are some limitations that must be addressed for reliable performance [101,102,103,104]. The advent of the eNose emerges as an encouraging development. The device’s capability for continuous adaptation through the assimilation of new samples enhances its prospects as a groundbreaking instrument for early and non-invasive diagnosis of PCa. A study conducted by Asimakopoulos et al. analyzed urine samples from 41 men subject to prostate biopsies, revealing a sensitivity of 71.4% and specificity of 92.6% [105]. However, the study’s control group consisted of patients with a single negative biopsy, which fails to definitively categorize a patient as PCa-free [105]. Similarly, Roine et al. tested an eNose on a group of 50 patients, yielding a sensitivity of 78% and a specificity of 67% in distinguishing between PCa and benign prostatic hyperplasia (BPH) [106]. The study acknowledged limitations in sample size and reliability of the control group as they were considered PCa-free solely based on negative histology after transurethral resection of the prostate (TURP).

**Table 3:** Summary of human urine studies evaluating eNoses for PCa detection. The table highlights key methodological features that may influence the interpretation of reported diagnostic performance, including control-group composition, blinding procedures, separation of training and test cohorts, validation strategy, and principal study limitations.

Study	System, Sample	Cohort, n	PCa Cases, n	Controls	csPCa	Blinding	Training/Test Split	External Validation	Main Limitations
Asimakopoulos et al., 2014 [105]	eNose urine headspace; initial/midstream urine	41	14	Biopsy-negative men	No	Not clearly reported	No	No	Small pilot cohort; single-center design; no independent testing; no csPCa endpoint; possible false-negative biopsy controls.
Roine et al., 2014 [106]	ChemPro 100 eNose; urine headspace	65 patients/74 samples	50	BPH patients undergoing TURP	No	Not clearly reported	LOOCV only	No	Small BPH control group; repeated control samples; no independent test set; no csPCa endpoint.
Aggio et al., 2016 [107]	GC-sensor system with LDA/SVM; urine headspace	155	58	Symptomatic non-cancer controls; bladder cancer also analyzed	Partial, Gleason reported	Not clearly reported	Internal validation only	No	Single-region pilot cohort; heterogeneous controls; risk of optimistic performance from internal model development.
Bax et al., 2022 [104]	Prototype eNose with drift compensation; urine headspace	122 (83 model subset)	81 (59 model subset)	Non-PCa controls	No	Double-blind reported	Yes	Temporal independent test set only	Small independent test set; limited controls; single-platform development; no multicenter validation; no csPCa endpoint.
Taverna et al., 2022 [92]	Prototype eNose with random forest; urine headspace	174	88	Healthy and non-PCa disease controls	Partial	Blind prospective	No clear external split	No	Single-cohort validation; heterogeneous controls; clinical role in biopsy triage not established.
Filianoti et al., 2022 [108]	Cyranose C320 eNose; urine headspace	272	133	Healthy controls	No	Not clearly reported	Cross-validation/internal repeated sampling	No	Enriched case–control design; healthy rather than clinical controls; limited biopsy-triage applicability.
Talens et al., 2023 [109]	MOOSY-32 eNose with neural-network classifier; urine	40 (800 generated files)	20	BPH patients	No	Not clearly reported	ML training/classification	No	Very small patient cohort; repeated files may inflate performance; limited clinical design details; no csPCa endpoint.
Taverna et al., 2024 [110]	Prototype eNose for PCa risk stratification; urine odor	120 validation cohort; 329 training cohort	120 validation; 329 training	No non-cancer controls	Yes, risk stratification	Blind prospective	Yes	Independent validation for risk stratification only	Not diagnostic vs. controls; all patients had PCa and underwent RARP; limited generalizability to screening/biopsy-triage settings; larger multicenter validation needed.

Abbreviations: BPH, benign prostatic hyperplasia; eNose, electronic nose; GC, gas chromatography; LDA, linear discriminant analysis; LOOCV, leave-one-out cross-validation; PCa, prostate cancer; RARP, robot-assisted radical prostatectomy; SVM, support vector machine; TURP, transurethral resection of the prostate.

Aggio et al. deployed a combination of gas chromatography with Metal Oxide Semiconductor (MOS) gas sensor to achieve high accuracy in detecting urological malignancies involving 58 men with PCa, 24 with bladder cancer and 73 with hematuria and or poor stream, without cancer, although the study reduced the reliability of its control group by not focusing on specific urological tumors [107]. A recent study by Talens et al. [109] investigated the development of a diagnostic tool for PCa using the Multisensory Odor Olfactory System MOOSY-32, an electronic nose system enhanced with a neural network. The approach was evaluated through the analysis of urine samples obtained from patients with both PCa and benign prostatic hyperplasia. The neural network, after being trained on this dataset, demonstrated a high degree of accuracy in differentiating between the two conditions, achieving a recall rate of 91% for detecting PCa. The training of the eNose remains a pivotal factor for achieving optimal performance [111], as does the stabilization of its classification abilities over time, enabled by specific compensatory models like drift compensation for the extensive wear and usage of the sensors, effectively mitigating sensor “aging” [104,111]. Recent evaluations of a new eNose variation have shown its high diagnostic accuracy for PCa detection in urine samples [92]. A comparative study by Grizzi et al. [112] found that eNoses demonstrated considerable effectiveness in PCa detection by comparing them with trained dogs which exhibited high accuracy in detecting PCa-specific VOCs in urine samples, thereby highlighting the potential of eNose integration into clinical practices, with promising implications for patient health in terms of early and non-invasive PCa diagnosis. Further research is needed to design well-structured clinical trials that can validate the integration of eNose technologies into diagnostic nomograms for PCa. In addition to diagnostic applications, recent evidence suggests that eNose technology may also play a significant role in PCa risk stratification. PCa is characterized by a highly heterogeneous clinical behavior, ranging from indolent forms to aggressive disease, making accurate stratification essential to guide therapeutic decision-making and avoid overtreatment. Current risk classification systems, such as the D’Amico model, categorize patients into low-, intermediate-, and high-risk groups based on clinical and pathological parameters; however, these approaches rely heavily on invasive procedures such as prostate biopsy and may suffer from sampling errors and limited accuracy. In this context, Taverna et al. [110] demonstrated that an eNose analyzing urinary VOCs is capable not only of detecting PCa but also of stratifying patients according to tumor aggressiveness. Specifically, the device achieved an accuracy of 74.2% when compared with the D’Amico classification and 79.2% when compared with pathological grade, with even higher performance in binary stratification scenarios (e.g., low vs. intermediate/high risk), reaching accuracies above 85–90%. Moreover, a substantial agreement was observed between eNose predictions and established risk models, including ISUP, CAPRA, and NCCN classifications. These findings highlight the potential of eNose as a non-invasive, rapid, and cost-effective tool not only for early diagnosis but also for pre-biopsy risk assessment and patient stratification. Such an approach could significantly improve clinical workflows by enabling more personalized management strategies and reducing unnecessary invasive procedures.

## 5. AI and the Diagnosis of Prostate Cancer

### 5.1. General Aspects of Artificial Intelligence

AI plays a central role in biomedical research and healthcare, particularly in the analysis of complex and high-dimensional datasets [113,114]. Its biomedical application has expanded rapidly with advances in computational methods and the increasing availability of digital medical data [115,116]. Among AI methodologies, machine learning (ML) is one of the most widely used approaches for identifying patterns in large datasets and developing predictive models for classification or outcome estimation [117,118,119]. In VOC-based diagnostics, ML is particularly relevant because eNose platforms and VOC-profiling technologies generate multidimensional signals that require computational processing before clinical interpretation. Feature selection is therefore essential to identify the most informative variables, including sensor-response descriptors, metabolomic signatures, and VOC-related patterns [120,121]. Deep learning (DL) may further support the analysis of complex non-linear relationships by automatically extracting hierarchical features from data [121,122,123], although its use in VOC- and eNose-based studies must be balanced against cohort size, interpretability, and the risk of overfitting. ML techniques include supervised approaches, which use labeled datasets for classification or prediction, and unsupervised approaches, which identify hidden patterns in unlabeled data [124,125,126,127,128,129,130,131,132]. Commonly applied algorithms include support vector machines, logistic regression, decision trees, random forests, artificial neural networks, clustering methods, and dimensionality-reduction techniques [127,128,129,130,131,132,133,134,135,136,137,138] (Table 4). Ensemble learning strategies, including bagging, boosting, and stacking, may improve predictive performance and robustness [130,139,140,141,142], but their application to VOC-based diagnostics requires rigorous train–test separation, prevention of data leakage, and external validation.

### 5.2. The Role of Artificial Intelligence in Prostate Cancer Detection

**Figure 4:**
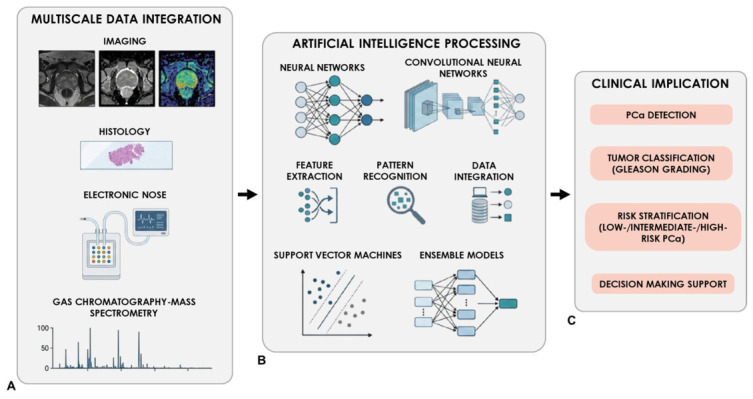
Schematic representation of AI applications in PCa. (**A**) illustrates VOC-based data sources, including urinary or breath VOC profiles obtained using eNose systems or GC–MS-based approaches. Additional clinical information, such as PSA levels, mpMRI findings, histopathological data, and risk-classification parameters, may provide complementary context in future multimodal models. (**B**) depicts the AI processing pipeline for VOC/eNose data, including sensor-signal preprocessing, feature extraction, feature selection, dimensionality reduction, pattern recognition, and classification using ML algorithms such as SVM, NN, LR, RF, and ensemble methods. Attention is required to ensure correct train–test separation, prevent data leakage, monitor sensor drift, support calibration transfer, and validate models externally. (**C**) summarizes potential clinical outputs, including PCa detection, identification of clinically significant disease, risk stratification, and decision support. Despite promising results, VOC/eNose-based AI systems remain investigational and require standardized protocols, prospective validation, explainability, and regulatory evaluation before clinical implementation.

Although AI has been widely explored in PCa imaging and histopathology, including mpMRI analysis, lesion detection, risk stratification, and automated Gleason grading [143,144,145,146,147,148,149,150,151,152,153], the focus of this review is its application to VOC-based diagnostics and eNose-derived sensor signals. Unlike imaging or histopathological data, VOC analysis and eNose platforms generate multidimensional chemical or sensor-response patterns that require dedicated computational workflows, including signal preprocessing, feature extraction, feature selection, dimensionality reduction, classification, validation, and clinical interpretation. In VOC-based PCa detection, ML algorithms can be used to identify disease-associated volatile patterns, distinguish PCa-related odor signatures from control profiles, and support the development of predictive models for sample classification. Commonly applied algorithms for VOC and eNose data classification are summarized in Table 4. These approaches are particularly relevant because eNose systems do not usually identify individual molecules, as GC–MS does, but rely on global sensor-response patterns generated by complex VOC mixtures. Therefore, the diagnostic output of an eNose depends not only on the sensor array itself, but also on the quality of the computational pipeline used to process and interpret the acquired signals. A critical step in AI-assisted VOC analysis is preprocessing of sensor signals. eNose responses may be influenced by baseline variability, instrumental noise, humidity, temperature, sample handling, storage conditions, and batch effects. Therefore, preprocessing may include baseline correction, normalization, smoothing, removal of unstable signals, alignment of response curves, and correction for technical variability. These procedures are essential because ML models may otherwise learn differences related to sample processing, environmental conditions, or instrument behavior rather than PCa-associated VOC signatures. Feature extraction and feature selection are equally important. Sensor arrays generate multidimensional responses, and each sensor may provide several informative descriptors, including maximum response, response slope, recovery time, area under the curve, steady-state response, or dynamic signal parameters. However, in many VOC/eNose studies, the number of extracted features may be high relative to the number of included patients. This increases the risk of overfitting, especially in small case–control cohorts. For this reason, dimensionality reduction and feature-selection procedures should be carefully implemented and transparently reported. A key methodological requirement is correct separation between training and testing datasets. Feature selection, normalization, dimensionality reduction, model tuning, and threshold optimization should be fitted only on the training set and then applied to the test set. If these steps are performed before train–test separation, information from the test set may indirectly influence model development, resulting in data leakage and artificially inflated estimates of sensitivity, specificity, accuracy, or area under the curve. Similarly, replicate measurements, repeated technical runs, or multiple samples from the same patient should not be divided between training and test datasets. Otherwise, the model may recognize patient-specific, batch-specific, or instrument-specific patterns rather than generalizable disease-associated VOC profiles. Several studies have investigated eNose technologies for PCa detection through urinary VOC analysis. These investigations report encouraging diagnostic performances, with sensitivities ranging approximately between 70 and 80% and specificities reaching values above 90% in some cases. More recent approaches have further improved diagnostic performance through the integration of AI algorithms, particularly neural network-based classifiers, which allow the analysis of complex multidimensional sensor responses. These AI-enhanced systems have demonstrated high performance in distinguishing PCa from benign conditions, achieving detection rates exceeding 90% in some studies [109]. Recent evaluations of novel eNose platforms have also reported promising results in detecting PCa through urinary VOC analysis [85]. Comparative studies have examined the performance of artificial olfaction systems relative to biological detection models, and Grizzi et al. showed that eNose systems can effectively identify PCa-specific VOC signatures in urine samples, achieving diagnostic performances comparable to those obtained with trained detection dogs [112]. Despite these promising findings, results should be interpreted with caution. Many studies evaluating VOC-based detection methods, including eNose platforms and biological olfactory models, are based on relatively small and often enriched case–control cohorts, which may not reflect real-world clinical populations. Control groups may include healthy volunteers, patients with benign prostatic hyperplasia, individuals with negative biopsies, or patients with other urological conditions, making comparisons across studies difficult. In addition, variability in sample collection, storage conditions, analytical protocols, sensor platforms, and model-training procedures may significantly influence classification performance. These issues are particularly relevant for VOC-based ML models, where differences in pre-analytical conditions, cohort composition, and data preprocessing may strongly affect the final diagnostic output [145,154,155,156,157,158,159]. External validation is therefore essential. A model developed on one cohort may perform well during internal validation but fail when applied to samples collected at another institution, processed at another time, or analyzed using a different sensor platform. Robust validation should ideally include independent, prospective, multicenter cohorts, representative of the intended clinical-use population. This is particularly important because many studies report diagnostic accuracy for the detection of “any PCa” without distinguishing between indolent and clinically significant disease. From a clinical perspective, the most relevant endpoint is the detection of clinically significant PCa, commonly defined as ISUP Grade Group ≥ 2. The ability of VOC-based approaches to reliably identify clinically significant disease, rather than low-risk tumors, remains insufficiently validated and represents a critical requirement for future clinical translation. Another major challenge associated with eNose systems is sensor drift, a phenomenon in which sensor responses progressively change over time because of sensor aging, repeated exposure to volatile compounds, contamination, environmental fluctuations, humidity, temperature, or calibration instability. Sensor drift can significantly affect the stability of classification models. AI techniques, including adaptive ML algorithms and drift compensation models, have been developed to mitigate these effects and maintain reliable diagnostic performance over extended periods of use [104,160]. However, their application requires systematic quality control, reference samples, periodic calibration, and longitudinal monitoring. Calibration transfer is closely related to sensor drift and represents another key issue for clinical translation. A model developed using one eNose device, sensor batch, or laboratory setup may not perform adequately on another device, even when the same sample type is analyzed. This limitation is particularly important for multicenter studies and future routine clinical use. Harmonized acquisition protocols, shared reference standards, calibration-transfer procedures, and multicenter validation datasets are therefore required to ensure that VOC-based classifiers remain stable across devices, institutions, and time. From a regulatory perspective, the sensor platform, preprocessing pipeline, calibration procedure, and ML classifier should be considered as components of a single integrated diagnostic system. Explainability is also important for clinical acceptance. Many AI models function as “black boxes”, limiting interpretability in clinical decision-making [161,162]. In VOC/eNose diagnostics, explainability may involve identifying which sensors, response phases, or extracted features contribute most strongly to classification. Feature-importance analysis, loading plots, sensitivity analysis, and model-agnostic interpretability tools may help determine whether a classifier is driven by biologically plausible VOC-related patterns or by confounding technical variables. This is essential for building clinical confidence and for supporting future regulatory evaluation. Yoo et al. [163] presented a urine-based diagnostic platform that integrates a six-member human olfactory receptor nanodisc sensor array with fluorescence detection and ML to identify PCa-associated VOCs. Sensor performance was first established in a curated cohort and subsequently validated in an expanded dataset, where ML classifiers achieved high diagnostic accuracy (0.890) and AUC (0.964), with key contributions from OR2W1, OR51E1, and OR51E2 olfactory receptors genes. The observed receptor response patterns correlated more strongly with Gleason score than with PSA levels, supporting the ability of this approach to capture tumor-specific metabolic signatures and suggesting potential applicability for non-invasive PCa detection. The AI workflow for VOC/eNose-based PCa detection includes signal acquisition, preprocessing, feature extraction, model development, validation, and potential clinical outputs (Figure 4). At present, PCa detection remains primarily based on PSA testing, risk-adapted strategies, mpMRI-guided pathways, and histopathological confirmation when biopsy is performed. Serum and urine biomarkers may be used as complementary tools in selected patients. In contrast, VOC-based technologies, including eNose platforms, ML-assisted VOC classification, and biological olfactory models, remain investigational. Their clinical utility in screening, biopsy indication, or risk stratification has not yet been established. Therefore, while the integration of AI with VOC-based sensing technologies offers promising perspectives for non-invasive PCa detection, further large-scale prospective studies, standardized methodological frameworks, external validation, sensor-drift control, calibration-transfer strategies, explainability, and regulatory alignment will be essential before clinical implementation can be considered.

## 6. Conclusions

PCa remains a major global health challenge, characterized by high incidence and significant clinical heterogeneity. While the widespread implementation of PSA-based screening has enabled earlier detection and contributed to reductions in advanced disease and mortality, it has also introduced substantial limitations, particularly in terms of overdiagnosis and overtreatment. The inability of PSA testing to reliably distinguish between indolent and clinically significant tumors underscores the urgent need for more precise, non-invasive, and clinically actionable diagnostic tools. Current diagnostic pathways, which integrate imaging modalities such as mpMRI with histopathological evaluation, represent significant advancements; however, they are still constrained by issues including interobserver variability, accessibility, cost, and the risk of missed or under-graded disease. In this evolving landscape, biomarkers, ranging from molecular assays to advanced imaging techniques, have emerged as valuable adjuncts for improving risk stratification and guiding clinical decision-making. Nevertheless, many of these tools remain limited by variability in performance, lack of standardization, and insufficient large-scale validation. As a result, there is growing interest in innovative diagnostic paradigms that can overcome these challenges while minimizing patient burden.

Volatolomics, the study of VOCs produced by metabolic processes, represents a particularly promising frontier in PCa diagnostics. The identification of disease-specific VOC signatures in biological fluids such as urine offers the potential for truly non-invasive, rapid, and cost-effective screening approaches. Importantly, VOC profiles reflect underlying biochemical alterations associated with tumor development and progression, providing a functional readout of disease biology that may complement traditional genomic and proteomic markers.

Recent advances in urinary VOC analysis are converging toward a coherent, non-invasive approach for PCa detection, supported by parallel developments in analytical chemistry, sensor technology, and computational modeling. Methodological innovations, such as solid-phase microextraction coupled with portable GC-MS and optimized sorbent-based workflows, have improved sensitivity, repeatability, and point-of-care feasibility, frequently achieving diagnostic performance above 80% [164,165]. At the same time, ML approaches applied directly to high-dimensional chromatographic data enable the extraction of disease-specific “scent signatures” without requiring full compound identification, reaching performance comparable to trained biological detectors and surpassing traditional PSA-based models [166,167]. These computational strategies integrate naturally with emerging biosensing paradigms, where organism-level responses, from trained canines to *C. elegans*, are decoded through AI to enhance diagnostic resolution and move beyond binary classification [63,168,169]. Clinically, metabolomic profiling studies have identified extensive panels of urinary VOCs capable of distinguishing cancer presence, aggressiveness, and post-treatment states, with some models achieving high accuracy and AUC values approaching or exceeding 0.9, although large-scale validation remains limited [170,171,172].

The current evidence on olfactory-based diagnostic approaches is limited by small, often enriched case–control cohorts, heterogeneous control populations, and variability in study design, including differences in sample handling and limited blinding. As a result, reported diagnostic performance metrics should be interpreted cautiously and are not directly comparable to established guideline-recommended tools, particularly when the clinically relevant endpoint is clinically significant PCa (ISUP Grade Group ≥ 2), rather than any PCa diagnosis.

Complementary sensor-based technologies, including eNoses, ion mobility spectrometry, MOS, and surface acoustic wave devices, further demonstrate the feasibility of rapid, non-invasive detection and risk stratification, in some cases reporting accuracies near 90%, while also highlighting challenges related to environmental interference and reproducibility [110,173,174,175].

Across platforms, integration of GC-based techniques with advanced statistical or ML models consistently improves discrimination between PCa, benign conditions, and other malignancies, reinforcing the concept of VOC patterns as disease-specific signatures [176,177]. Mechanistically, these signatures reflect tumor-driven metabolic alterations involving lipid peroxidation and related biochemical pathways, producing characteristic compounds such as aldehydes, ketones, and aromatics [32,178,179]. Systematic and narrative reviews confirm that VOC-based diagnostics across cancers commonly achieve sensitivities and specificities in the 70–90% range, while emphasizing persistent limitations including cohort heterogeneity, lack of standardized protocols, and variability in analytical methods [180,181,182]. Additional epidemiological and cross-matrix studies suggest that VOCs not only serve as diagnostic biomarkers but may also reflect environmental exposures and broader metabolic states, further expanding their relevance in oncology [183]. The field is transitioning from single-analyte approaches toward integrated, pattern-based diagnostics that combine chemical profiling, sensor technologies, and AI, with strong potential for early detection, risk stratification, and monitoring of PCa, although rigorous standardization and large-scale clinical validation remain essential for routine implementation.

Biological olfactory systems, including those of nematodes and dogs, have demonstrated remarkable sensitivity and specificity in detecting PCa-associated VOCs. The development of these new study models is closely tied to restrictions on vertebrate animal experimentation, which have encouraged researchers to reduce their use and adopt alternative models whenever possible. Organisms such as *C. elegans* offer unique advantages, including rapid training, scalability, and the ability to detect subtle metabolic differences that may be difficult to identify using conventional analytical methods. Similarly, trained detection dogs have demonstrated high diagnostic accuracy in identifying PCa from urine samples, underscoring the robustness of olfactory-based detection.

The use of animals in diagnostic research raises important ethical concerns, particularly when it involves mammals such as dogs, mice, and rats. Dogs show high sensitivity and specificity and can analyze many samples over several years, but their use is limited by long training times, high costs, and the requirement for ethical approval. Similarly, rodents require ethical documentation and often large numbers of individuals, despite benefiting from short training periods and good diagnostic performance. In contrast, non-mammalian models represent a more ethically acceptable option. These organisms are available in large numbers, require little or no training, and enable rapid testing within minutes or hours. Many of these models also do not require formal ethical approval, facilitating their use in experimental settings. Although limitations remain, such as the need for multiple individuals per test or reduced discrimination in some species, these simpler organisms offer a practical and more ethically favorable alternative to mammalian detectors in cancer research. However, despite their impressive performance, the clinical translation of these biological systems is limited by issues related to standardization, reproducibility, regulatory approval, and logistical constraints. To address these limitations, significant efforts have been directed toward the development of artificial olfactory systems, such as eNoses, which aim to replicate the pattern-recognition capabilities of biological sensors. eNose technology offers several advantages, including portability, rapid analysis, and potential integration into clinical workflows. When combined with advanced ML algorithms, these systems can analyze complex VOC mixtures and achieve promising diagnostic performance in distinguishing PCa from benign conditions, as well as in risk stratification. Technical challenges such as sensor drift, environmental sensitivity, and the need for robust calibration remain important barriers to widespread clinical adoption.

AI further enhances the potential of these emerging technologies by enabling the analysis of high-dimensional datasets derived from imaging, histopathology, and VOC-based platforms. AI-driven models have demonstrated the ability to improve diagnostic accuracy, reduce variability, and support clinical decision-making across multiple stages of the PCa diagnostic pathway. Nonetheless, challenges related to model interpretability, data bias, and the need for large, diverse training datasets must be carefully addressed to ensure safe and equitable implementation.

The future of PCa diagnostics is likely to be shaped by a multimodal approach that integrates traditional clinical tools with innovative technologies such as VOC analysis, bio-inspired sensing systems, and AI-driven analytics. While considerable progress has been made, further research is essential to validate these approaches in large, prospective clinical studies, establish standardized protocols, and ensure regulatory approval.

However, it is important to emphasize that current clinical pathways for PCa detection remain centered on PSA-based screening, risk-adapted strategies, and the use of mpMRI as a triage tool prior to biopsy, with additional biomarkers used only in selected cases. In contrast, VOC-based approaches, including eNose systems, canine olfaction, and invertebrate models, are not currently endorsed by clinical guidelines and should be considered investigational. At present, these technologies cannot be used for routine screening, biopsy decision-making, or risk stratification, and their role should be regarded as complementary and exploratory, pending further validation.

Ultimately, the successful translation of these innovations into clinical practice has the potential to significantly improve early detection, reduce unnecessary interventions, and enable more personalized and effective management of PCa.

In conclusion, VOC-based diagnostics combined with advanced sensing technologies and AI-driven analytics represent a promising and rapidly evolving frontier in PCa detection. Since no universal cancer biomarker has been identified and each cancer type likely exhibits a distinct biomarker pattern rather than a single shared molecule, capturing disease-related metabolic signatures in a non-invasive manner offers a valuable approach. These methods have the potential to complement existing diagnostic pathways and improve risk stratification; however, their clinical integration will depend on rigorous validation, standardization, and demonstration of robust performance across large, diverse patient populations.

## 7. Patents

Taverna G., Grizzi F., Capelli L., Sironi S., Bax C., and Eusebio L. METHODS TO ASSESS THE RISK OF BEING AFFECTED BY PROSTATE CANCER. International application number PCT/EP2020/055555; International filling date 3 March 2020. (Humanitas Mirasole S.p.A. 60%, Politecnico di Milano 40%).

## Figures and Tables

**Table 1:** Summary of *C. elegans*-based olfactory studies relevant to prostate cancer detection. For each study, the biological system and sample type, cohort size, number of prostate cancer cases, definition of the control group, assessment of csPCa, blinding procedures, separation of training and test cohorts, external validation, and principal methodological limitations are reported.

Study	System, Sample	Cohort, n	PCa Cases, n	Controls	csPCa	Blinding	Training/Test Split	External Validation	Main Limitations
Thompson et al., 2021 [63]	*C. elegans* Bristol N2 chemotaxis assay; urine	67	21	Benign/suspected PCa with negative biopsy; negative-screen controls	Partial	Partial	No	No	Small single cohort; mixed controls; partial blinding; no independent test set; csPCa not prospectively validated; possible optimism from within-study dilution optimization.
Hatakeyama et al., 2024 [56]	N-NOSE *C. elegans* chemotaxis assay; urine	221	159	No concurrent non-cancer control group	No	Not clearly reported	Not applicable/predefined threshold	No PCa-specific validation	Not PCa-specific; no specificity estimates in cohort; no comparison with PSA, MRI, biopsy indication, or BPH controls.

Abbreviations: BPH: benign prostatic disease; csPCa: clinically significant prostate cancer; PCa, prostate cancer; PSA, prostate-specific antigen; MRI: magnetic resonance imaging; N-NOSE: Nematode-NOSE.

**Table 2:** Summary of human urine studies evaluating trained canine olfaction for PCa detection. The table highlights key methodological features that may affect the interpretation of reported diagnostic performance, including control-group composition, blinding procedures, separation of training and test cohorts, validation strategy, and principal study limitations.

Study	System, Sample	Cohort, n	PCa Cases, n	Controls	csPCa	Blinding	Training/Test Split	External Validation	Main Limitations
Gordon et al., 2008 [76]	Canine detection of urinary odor	11	11	Age- and sex-matched healthy volunteers	No	Limited/late	Partial	No	Very small PCa sample; healthy rather than clinical controls; limited standardization; poor sensitivity; not clinically translatable.
Cornu et al., 2011 [77]	Single trained dog; urine from men referred for PSA/DRE abnormalities	66	33	Biopsy-negative men with elevated PSA and/or abnormal DRE	Partial	Double-blind	Yes	No	Single dog; small-enriched cohort; csPCa not primary endpoint.
Elliker et al., 2014 [78]	Double-blind canine discrimination study using urine	117;	50	BPH patients and healthy men	No	Double-blind	Yes	No	Limited training samples; poor generalization to unfamiliar samples; controls not fully representative of biopsy-negative diagnostic populations.
Urbanova et al., 2015 [79]	Single trained dog; urine samples	70	45	Urological patients with negative histology	No	Not clearly reported	Partial	No	Single dog; modest cohort; unclear blinding; no independent validation; no csPCa endpoint.
Taverna et al., 2015 [80]	Two trained dogs; cross-sectional urine diagnostic study	902	362	Healthy subjects, non-neoplastic diseases, non-prostatic tumors	Partial	Blinded	Structured training/testing	No	Enriched cohort; controls not limited to biopsy-negative suspected PCa; limited real-world biopsy-decision applicability.
Guest et al., 2021 [81]	Double-blind pilot integrating canine olfaction with GC-MS, ANN, and microbiota profiling	50 (28 in blind trial)	12 (7 in blind trial)	Biopsy-negative controls	Indirectly, Gleason 9 only	Double-blind	Yes	No	Very small high-grade PCa test set; recalibration required; feasibility design only; no definitive validation.
Hermieu et al., 2024 [82]	Prospective double-blind validation in men undergoing biopsy	151	78	Negative biopsy or ISUP 1	Yes, ISUP ≥2	Double-blind	Yes	No independent external validation	Inter-dog variability; lower validation than training performance; possible biopsy-label uncertainty; no long-term outcome validation; limited scalability.

Abbreviations: ANN, artificial neural network; BPH, benign prostatic hyperplasia; csPCa: clinically significant prostate cancer; DRE, digital rectal examination; GC-MS, gas chromatography-mass spectrometry; ISUP, International Society of Urological Pathology; PCa, prostate cancer; PSA, prostate-specific antigen.

**Table 4:** Overview of commonly used ML algorithms for VOC analysis and eNose data classification. The table summarizes representative algorithms, their acronyms, and their main computational principles as applied to pattern recognition, dimensionality reduction, and classification of complex sensor-derived signals.

Algorithm	Acronym	Description
Logistic Regression	LR	Statistical classification method used for binary or multi-class prediction, estimating the probability of class membership based on input features.
Support Vector Machine	SVM	Supervised learning algorithm that identifies the optimal separating hyperplane between classes, particularly effective in high-dimensional datasets such as VOC profiles.
Random Forest	RF	Ensemble learning method based on multiple decision trees, improving classification accuracy and robustness while reducing overfitting.
k-Nearest Neighbors	k-NN	Non-parametric method that classifies samples based on the majority class of their nearest neighbors in feature space.
Artificial Neural Network	ANN	Computational model inspired by biological neural networks, capable of learning complex nonlinear relationships in multidimensional data.
Principal Component Analysis	PCA	Unsupervised dimensionality reduction technique used to identify patterns and visualize variability in complex datasets such as VOC signals.
Linear Discriminant Analysis	LDA	Supervised method used for dimensionality reduction and classification, maximizing the separation between predefined classes.

## Data Availability

No new data were created or analyzed in this study. Data sharing is not applicable to this article.
